# The application of the QALY measure in the assessment of the effects of health interventions on an older population: a systematic scoping review

**DOI:** 10.1186/s13690-021-00729-7

**Published:** 2021-11-18

**Authors:** Ewa Kocot, Paulina Kotarba, Katarzyna Dubas-Jakóbczyk

**Affiliations:** 1 Health Economics and Social Security Department, Institute of Public Health, Faculty of Health Sciences, Jagiellonian University Medical College, Krakow, Poland; 2 Department of Health, Małopolska Provincial Office in Krakow, Krakow, Poland

**Keywords:** Quality adjusted life year (QALY), Older population, Resource allocation

## Abstract

**Background:**

One of the most commonly used types of evaluation methods is cost-utility analysis (CUA), using the Quality Adjusted Life Year (QALY) indicator as a preference-based measure for assessing effects of a given programme. Such assessments are often translated into health-care provision priorities; therefore, effectively choosing the method of outcome evaluation is crucial for ensuring the best possible allocation of scarce resources. The main objective of this scoping review is to identify what kinds of problems and limitations may occur when the QALY indicator is used to assess the effects of health interventions in the older population.

**Methods:**

To identify literature in a scoping review, the databases MEDLINE via PubMed and Scopus were searched. A manual search on relevant organizations’ and associations’ websites was also conducted (EUnetHTA, ISPOR and national governmental agencies responsible for allocation decisions). No limits concerning publication dates were set. All relevant data were extracted and analyzed, then a narrative summary was prepared.

**Results:**

The database search identified 10,832 relevant items, finally 32 studies were included in the analysis. The main types of issues indicated in the studies were as follows: (1) lower life expectancy in the older population causes lower QALY gains; (2) an equal value of one QALY is used regardless of age; (3) poorer average health state causes lower QALY gains; (4) inadequate instruments to measure quality of life (QoL); (5) attributes of QoL used regardless of age; and (6) no beyond-health QoL aspects taken into account.

**Conclusions:**

This review shows clearly that many problems of different types are connected with using QALY for the older population, but there is no consensus as to whether QALY discriminates against the older population or not – an opinion regarding this issue depends strongly on accepted principles, particularly the approach to equity and how one understands fairness. Health care resources should not be allocated solely on the basis of the health maximization rule because this can lead to discrimination against certain groups (e.g., older, disabled, and/or chronically ill people). To maintain the balance between efficiency and equity, the issues connected with age-based rationing should be widely discussed.

## Background

Resource allocation in the health care sector requires (and will require in the future) decision-makers to make difficult decisions about setting priorities. To provide evidence-based indications for making these decisions, economic evaluations comparing the costs and effects of various health programmes are increasingly prevalent [1, 2]. One of the most commonly used type of evaluation method is cost-utility analysis (CUA), using the Quality Adjusted Life Year (QALY) indicator as a preference-based measure for assessing effects of a programme. The main advantage of QALY is that it combines in a single indicator the measurement of quality of life (QoL) and the length of life. As QALY can be calculated for nearly every kind of treatment, intervention, or procedure, this indicator has a high comparative potential and is widely accepted as enabling comparison of health benefits [3]. The QALY measure is recommended or even mandatory in health technology assessment procedures in many countries [4]. It is used to assess the effects of health programmes in all age groups, from the youngest to the oldest. However, the question arises of whether the results presented in QALY are really fully comparable between populations of all ages, without any bias.

During the QALY calculation, both health status (in terms of QoL) and length of life are taken into account. In the case of older people, health status is usually worse and life expectancy is shorter than for younger people; so, looking at the QALY construction, it can be expected that the programme benefits assessed by this method will usually be valued lower for the old.

Such assessments are often translated into health-care provision priorities; therefore, effectively choosing the method of outcome evaluation is crucial for ensuring the best possible allocation of scarce resources. Traditionally, the lowest cost per QALY is an indication for prioritization with the aim of maximizing health gain in the population [5]. However, growing evidence shows that the rule of health gains maximisation is not sufficient when making allocative decisions. Equity and fairness issues are also important and equity weighing should be incorporated into allocative decisions as they may have enormous ethical consequences [5, 6]. From a societal point of view, some health gains (which can also be presented in the QALYs) can be considered to be more valuable for the general population than others [7]. The issue of how to reconcile different preferences in allocation decisions with traditional economic evaluation indications is still under debate [5, 7–9].

QALY has been broadly criticized in the literature due to many problems that arise during the calculation process (e.g. [10–15]). Among other things, the QoL assessment, an integral part of this process, requires a valuation of health states. However, perception of health and diseases may vary significantly between people and a lot of heterogeneity can emerge in this assessment. Additionally, there is no general consensus as to which dimensions of human life should be taken into account when assessing QoL, and considering all significant ones is not technically possible. There are also many problems regarding equity consideration when decisions are made in line with results of analyses based on QALY. Not all issues connected with QALY concern the older population specifically and they may impact the results of the economic evaluation differently for different age groups. The main objective of this research is to identify what kind of problems and limitations may occur when the QALY indicator is used to assess the effects of health interventions in the older population. To the authors’ knowledge, based on quick database reviews, no extended review related to this topic, and focused specifically on the older population, has been done so far.

## Methods

A scoping literature review was conducted, based on the methodological framework outlined by Peters et al. [16]. This project has been registered through the Open Science Framework.

### Research questions

To achieve the general objective of this study, three specific research questions were defined:

1. What type of research has been done to identify the relevant issues?
2. What kinds of problems can arise when applying the QALY indicator for the older population?
3. What solutions the identified problems have been proposed and what recommendations have been formulated?

### Search process

To identify literature, the databases MEDLINE via PubMed and Scopus were searched. A manual search on relevant organizations’ and associations’ websites was also conducted (EUnetHTA, ISPOR and national governmental agencies responsible for allocation decisions). Finally, the reference lists of included publications were scanned to identify interesting items. No limits concerning publication dates were set. The database searches were conducted on February 16th 2021. Internet websites of organizations and projects were screened in April 2021. The further analysis took place in May and June 2021.

There were two groups of search terms defined: (1) indicator (QALY) and (2) population (older). The Boolean operator “OR” was used between terms inside each group and the groups were then connected by the Boolean operator “AND”.

Two groups of search terms were defined:

1. **indicator**
  MEDLINE by PubMed: QALY OR “Quality Adjusted Life Year*” OR qaly [MeSH Terms].
  Scopus: ALL (QALY) OR ALL(“Quality Adjusted Life Year*”)
2. **population**
  MEDLINE by PubMed: “older population*” OR “older adult*” OR “older people” OR “older group*” OR elderly OR aged OR aged [MeSH Terms]) OR elderly [MeSH Terms]).
  Scopus: ALL (elderly) OR ALL(“older adults”) OR ALL(“older population”) OR ALL(“older people”) OR ALL (aged).

The groups were then connected by the Boolean operator “AND”; for Scopus search, the component NOT INDEX (medline) with AND operator was added.

### Selection process

The process of removing duplicates and further records management was conducted using the Mendeley bibliographic program. On both conducted stages of selection ((1) a title and abstract review; (2) a full-text review) the studies were assessed for eligibility independently by two authors. The third author was asked for an additional opinion in the case of conflicting decisions and the majority opinion was decisive.

In the selection process, the inclusion criteria were used as follows: (1) identified problems are related specifically to the QALY measure; (2) identified problems are older age-related; (3) the research applies to broader context, not case-study; and (4) the full text is available in English. The criteria to exclude a study were: (1) identified problems are not related to QALY; (2) identified problems are related to factors other than older age; (3) the research focuses on a specific case, with no generalization possible; (4) the full text is in a language other than English/no full text is available. In line with the methodological framework adopted, no selection was based on a quality assessment [16].

### Data extraction

The data extraction table was designed in accordance with the defined research question. The following fields were included: (1) Author and title; (2) publication year; (3) type of study/methodology used; (4) objective of study; (5) QALY use as a main subject of the study (Y/N); (6) type of problem with QALY indicated; (7) main results/findings; (8) conclusions; (9) recommendations/solutions proposed.

## Results

### Search results

**Fig. 1:**
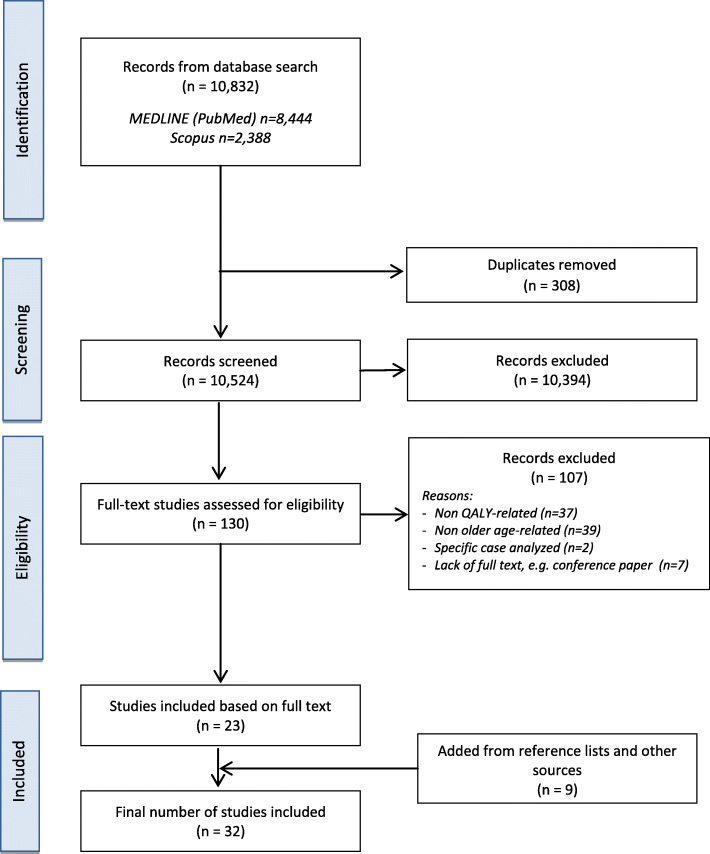
PRISMA Flow Diagram

The database search identified 10,832 relevant items. In the end, 23 of them and nine others from reference lists and other sources (32 in total) were included in the analysis as a result of the search process (Fig. 1).

### Overall characteristics of the included studies

About 40% of the included studies (*n* = 13; 40.6%) were published before the year 2000 (four of them before 1990, nine in the years 1990–1999), nine (28.1%) in the period 2000–2009 and ten (31.3%) between 2010 and 2018. No studies published after 2018 were found.

In a majority of the included studies (*n* = 21; 65.6%) the issues regarding the use of the QALY measure for the older population was not a main topic (or even one on the main topics) and comments regarding the topic of interest came up by way of analysing other more or less related problems, such as, for instance, the social value of QALY.

More than 40% of the analysed studies were original research (*n* = 13; 40.6%), mostly based on a conducted survey (*n* = 10; 31.3%). Almost the same number of publications (*n* = 12; 37.5%) were written as expert opinions, usually based on a more or less advanced argumentative literature review. Only three studies were literature reviews (systematic or narrative).

The problems of using the QALY for the older population indicated in the studies can be divided into six groups (Table 1). Only one of them is directly connected with the saving life year gain component of QALY, but this kind of problem was mentioned in more than half of the analysed studies (*n* = 17; 53.1%; problem 1 in Table 1). In 15 studies, the authors highlighted problems related to the quality of life component of QALY (*n* = 15; 46.9%; problems 3–6 in Table 1). In exactly half of the publications (*n* = 16; 50%; problem 2) an issue associated with an equal valuation of each QALY regardless of age was indicated (not related to the specific QALY component: QoL or length of life). This categorization of problems is used later in this article as a framework for issue analysis.

**Table 1:** Types of problems emerging when using QALY for the older population, indicated in the studies

Type of problem	Component relationship	N^**a**^	References
1.Lower life expectancy (LE) causes lower QALY gains ***(LE)***	YoL	17	[11–27]
2.Equal value of one QALY regardless of age ***(Q = Q)***	Non specific	16	[19, 20, 24, 26, 28–39]
3.Poorer average health state causes lower QALY gains in QoL component ***(QoL)***	QoL	8	[14, 17, 18, 26, 36, 39–41]
4.QoL measure instrument inadequacy ***(Instr.)***	QoL	7	[15, 17, 26, 31, 40, 42, 43]
5. Health-related QoL measured regardless of age, while different attributes/attribute measurements are important for older people ***(Attrib.)***	QoL	5	[20, 24, 38, 40, 44]
6.No beyond-health QoL aspects taken into account ***(B-HA)***	QoL	4	[14, 40, 45, 46]

^a^The sum is not equal to the number of publications as some of them include more than one type of problem

*YoL* years of life, *QoL* quality of life

**Fig. 2:**
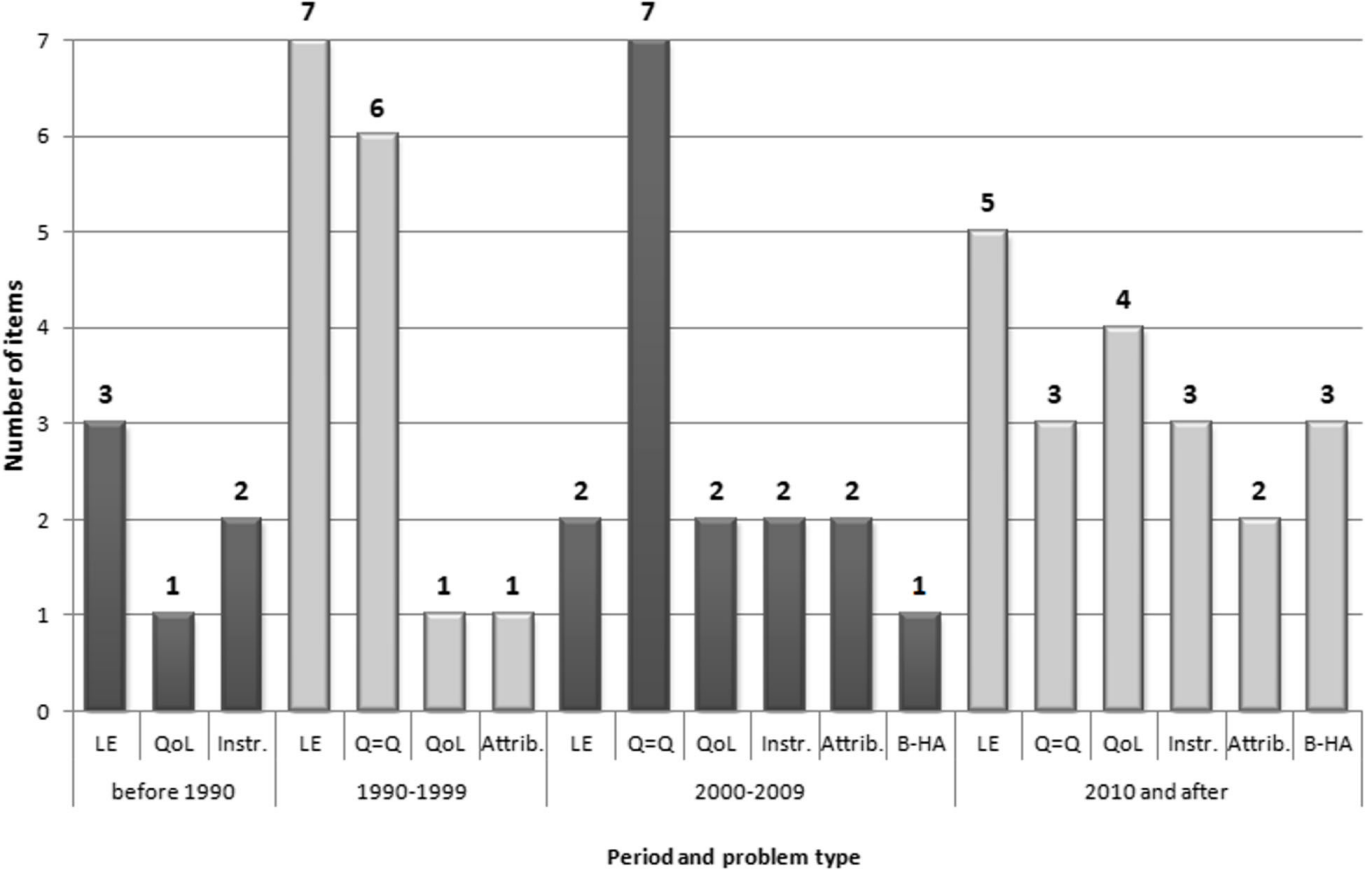
The type of problems indicated in the studies by years. *Notes: (1) The sum of items is not equal to the number of publications as some of them include more than one type of problem; (2) abbreviations of problem type are explained in* Table 1

Only three studies published before the year 2000 indicated problems related to the scope of the measured QoL or to instruments used for its valuation (two regarding instrument aspect (problem 4) and one about health-related QoL measured regardless of age (problem 5)). After the year 2000 these kinds of issues were much more widely discussed and highlighted in 13 publications. An issue associated with the year of life gains component is mentioned in most of the publications in each time period (Fig. 2).

The full list of included studies with more details regarding study characteristics can be found in Table 2.

**Table 2:** The characteristics of included studies

	Author(s) and year	Type of study	Is using QALY in older population a main topic?	Type of problem discussed
1	Asim & Petrou 2005 [38]	O/AR	N	QALY = QALY; Attrib
2	Avorn 1984 [17]	O/AR	N	LE; QoL; Instr
3	Baltussen et al.1996 [18]	Rev	P	LE; QoL
4	Blomqvist 2002 [42]	R	N	Instr
5	Brouwer et al. 2005 [39]	R(S)	N	QALY = QALY; QoL
6	Bulamu et al. 2015 [45]	Rev	N	B-HA
7	Busschbach et al. 1993 [32]	R(S)	Y	QALY = QALY
8	Cookson & Culyer 2010 [14]	O/AR	N	LE; QoL; B-HA
9	Dey & Fraser 2017 [26]	Rev	N	LE; QALY = QALY; QoL; Instr
10	Donaldson et al. 1988 [43]	R	N	Instr
11	Franklin 2017 [41]	O/AR	N	QoL
12	Grewal et al.2006 [46]	R(S)	N	B-HA
13	Harris 1987 [27]	O/AR	N	LE
14	Hazra et al. 2018 [28]	O/AR	N	LE; QALY = QALY
15	Huter et al. 2016 [40]	O/AR	N	LE; QoL; Instr; Attrib; B-HA
16	Huter et al. 2018 [44]	Rev	N	Attrib
17	Johannesson & Johansson 1996 [29]	R(S)	N	LE; QALY = QALY
18	Johannesson & Johansson 1997 [30]	R(S)	Y	LE; QALY = QALY
19	Johri et al. 2005 [31]	R(S)	P	LE; QALY = QALY; Instr
20	Joiner 1999 [19]	O/AR	Y	LE; QALY = QALY
21	Kappel & Sandoe 1992 [20]	O/AR	P	LE; QALY = QALY; Attrib
22	Kappel & Sandoe 1994 [21]	O/AR	P	LE
23	Mendeloff 1983 [22]	O/AR	N	LE
24	Nord et al. 1996 [23]	R(S)	N	LE
25	Petrou 2014 [33]	Rev	P	QALY = QALY
26	Pettitt et al. 2016 [15]	Rev	Y	Instr
27	Rodriguez & Pinto 2000 [34]	R(S)	Y	QALY = QALY
28	Stevens et al. 2012 [25]	Rev	P	LE
29	Stolk et al. 2005 [35]	R(S)	N	QALY = QALY
30	Tsuchiya 2000 [36]	R	N	QALY = QALY; QoL
31	Tsuchiya et al. 2003 [24]	R(S); Rev	N	LE; QALY = QALY; Attrib
32	Williams 1997 [37]	O/AR	N	QALY = QALY

Legend:

Type of study

*• O/AR*: authors’ opinions, also based on an argumentative review

*• R(S):* research based on survey

*• R*: other than survey research

*• Rev*: systematic or narrative literature review

Is using QALY in the older population a main topic?

*• N*: no

*• P*: it is one of the main topics of the study, but not the only

*• Y*: yes

Type of problem discussed

*• Attrib*.: QoL measured regardless of age, while different attributes/attribute measures are important for the older population

*• B-HA*: no beyond-health QoL aspects taken into account

*• Instr*.: problems with inadequacy of QoL measurement instrument

*• LE*: lower LE causes lower QALY gains

*• QALY = QALY*: equal value of one QALY regardless of age

*• QoL*: poorer average health state causes lower QALY gain

### Problems indicated in the studies

Below, the main findings contained in the included studies are described, according to the type of problem they relate to.

#### Lower LE causes lower QALY gains

This most frequently analysed problem (in 53% of the studies) was that young people undoubtedly have a longer life expectancy than older people. Therefore, in terms of the numbers of life years gained, programmes for the young always (or nearly always) have larger effects than programmes for the old [14, 17, 25]. This problem concerns not only QALY, but all measures based on added years of life. Young people simply have more life years to gain [19–23, 27, 29–31, 40]. This issue can be described as a “ceiling effect” and may bias results against older people [31]. In fact, looking at the concept, age per se is not a cause of potential bias here, but rather LE. However, lower LE is strongly connected with age [24].

Even if the efficacy of a given intervention is the same for the old and the young, the potential duration of effects is lower in older age, due to the lower remaining LE. But this is the case only for long life extension effects – if intervention benefits are relatively short-lasting, gains will be similar [18]. This problem may be of minor importance when an evaluated programme is not only directed at the older population, as in this case, average results for all age groups together are usually taken into account. But if a programme is predominantly for older patients, or analyses are done separately for different age groups, using QALY may lead to discrimination against older people [26].

QALYs will be gained from an intervention over a longer period for the young than for the old, but this issue relates mainly to interventions with short-term costs. For chronic conditions, costs are incurred over a long period of time, so a longer LE means higher costs as well as effects. Therefore, in this case, the discrimination in priority setting against the older population my not be present [28].

#### Equal value of one QALY regardless of age

Health in the general QALY concept is valued equally for all people, regardless of age or other individual characteristic. One QALY gained by a young patient is equal to one QALY gained by an old patient - each additional QALY is considered equal, so it is rather an egalitarian than a utilitarian approach [32, 36]. As 1 year of a given health is valued equally, formally speaking, QALY is not ageist [20]. However, there is a doubt as to whether one QALY should indeed be valued the same for the young as for the old [19, 24, 34, 35, 37, 38].

The equal valuation of QALY, regardless of age often seems inconsistent with general public preferences, as society would place different values on health gained at different stages of life: in general, the social value of health is often found to be higher for younger people than for older people [26, 29, 30, 32]. In line with this point of view, assuming equal QALY values can be a problem when comparing QALY gains for the young and the old.

Arguments against QALY equality in terms of age can be various. One of them is the “fair-innings” concept which assumes that every person should have an equal chance of living “a normal” life – meaning “normal” length and quality. As older people have already attained a larger share of their “fair innings”, the younger population should receive more health care currently. “Fair innings” assumes that the social value of health benefits is based not only on the efficiency criterion, but also distributive context matters [28, 31, 35, 36]. However, it is not a rule that people living to advanced ages used more health care earlier in life – they might have used a minimal amount, but expect to benefit more from the health system in older age. Living a “fair share” of life doesn’t always mean receiving a “fair share” of health care [28].

There is growing evidence that people give a higher value to health gains for the young, but many methodological constraints in research in this area still exist [33, 34]. The intervention type may also matter in terms of general public assessment: age is important when it comes to life-saving intervention, but much less important in terms of pain relief or depression [31].

One more argument against QALY equality for all is the level of acceptable health state. It may be varied for different ages, so one QALY when one is young is not the same as one QALY when one is old if the acceptable level of health (lower than ideal) is considered maximal for the latter [39].

The above may have consequences for QALY analysis: in general, people consider health of younger people to be more valuable than the health of older people, while in the general QALY concept, the value is weighted the same for each age group. On one hand, the QALY analysis creates differences based on effectiveness of treatment (which is often lower for older people), but on the other hand, it does not take into account differences between people and social preferences regarding it. QALY analysis can be seen then as more egalitarian than the general public prefers [32].

#### Poorer average health state causes lower QALY gains in the QoL component

Each method of evaluating gains in QoL may discriminate based on disability [26]. The quality of life is usually lower in older age (e.g., because of comorbidities), so even if the same number of years is saved for a young and an old person, using QALY methodology, the latter will be valued as less beneficial. An intervention, even when completely successful, does not improve the health of an older person with co-morbidities to the level of ideal health [14, 17, 40]. “Full health” is seen as full psychophysical function, but this can be problematic in the case of patients with a permanent disability and co-morbidities, which frequently emerge in older age. An intervention can only limit their disability, but „full health” state, as defined in the QALY methodology, is impossible to achieve. As a result, QALY gains for such people is evaluated lower [41]. A fully successful health intervention in the case of older people result in smaller QALY gains then for younger people, as the maximal possible QoL is lower, older people may respond less to a treatment, more complications occur, and the capacity to recover is simply limited [18, 36].

At different ages there can be different meanings of “good health”. A “perfect health equals 1” is used in the standard QALY calculation as a reference point for health gains. As a consequence, the fact that this ideal level of health is not possible (or is more often not possible) to reach in the case of older people may cause an incorrect estimation of health effects. People adapt to reduced health states as they become older and as they link their health assessment to that of similar groups rather than to perfect health, they are willing to accept a worse health state as “normal”. Research has confirmed that the acceptance of a lower than perfect level of health increases with age (with differences depending on the health domain: for instance, some health problems, such as reduced mobility, had the highest level of acceptability, while anxiety/depression was very often considered unacceptable even in older age). So from the societal perspective, the same health gain may be differently valued, depending on a given individual’s level of accepted health state [39].

For long-term care, more often provided to the older population, the goal would be to slow down the loss of QoL rather than to increase QoL - whereas for acute care, the goal is to improve it. This differentiation of the attitude to health interventions effects can be important when comparing effectiveness [26].

#### QoL measurement instrument inadequacy

Answering standard-gamble or similarly constructed questions individuals can take into account expected life-time budget constraints connected with a given health state. Young people are likely to accept a relatively large decrease in expected life years in order to compensate for a given decrease in quality, as they take into account the potential necessity of consumption reduction in the case of ill health when young (a reduction in youth and, consequently, in old age as well). A different approach of young and old to issues related to the potential financial consequences may result in a bias against older in allocation decisions [42].

Very ill people often value their QoL differently than healthy people asked to assess the same health state. For older groups, it is increasingly often not possible to use patient-based data to determine numerical values of health states, as many diseases (e.g., dementia) limit contact with patients [17]. Basing the quality assessment on a different type of data source can cause problems in comparability.

Long-term care, more often provided to older people, may have a minimal impact on health, and the potential for health gains is often limited in old age. If the instruments used to measure QoL changes are not sensitive enough to measure these small changes (as is often a case), the QALYs gain is small when compared to more acute forms of care, or even equal to zero [15, 26, 31, 40, 43].

#### Health-related QoL measured regardless of age

The QALY is linked to health-related QoL, valued on the basis of the preferences of a tested group of people and determined independently of any other features (like age, for instance) [20]. The QALY method assumes that changes in QoL caused by a given treatment are independent of age, but in fact, QoL resulting from the same health condition can differ for the young and the old. It is not clear whether for some age groups it is lower or higher – older people can value their health less, but on the other side, can also start to expect more from life and perceive good health as more important [20]. The research of Tsuchiya et al. indicates that some health problems can cause a larger loss of QoL to a younger person than to older person [24]. In any case, significant differences between the young and the old may emerge.

One QoL measure that is the same for all groups may not correspond to people’s experiences and expectations, as various factors/attributes influence health-related quality of life and do so in different ways. Some attributes of health-related QoL may become more or less important with increasing age, as preferences change in the course of life; e.g., mobility, crucial for the young, may be of less significance for older people, as a certain range of restrictions in this area is accepted and considered normal in old age. Therefore, a loss of QoL caused by the same bad health state and, accordingly, a gain of QoL obtained as a result of an intervention can be valued totally different at different ages. The measurement of QoL for older people needs to be more focused on the health-related ability to undertake the activities of daily living and the capability to achieve different functions. The currently used preference-based utility measures are not in line with the real needs of older people [38, 40, 44].

#### No beyond-health QoL aspects taken into account

The instruments used to assess the QoL component in QALY calculation limit this assessment to dimensions related to health-related QoL. Whereas, for old people, aspects other than those related to health are often essential. It is sometimes difficult to separate health needs from social needs in the older population. Moreover, social values (integration into the community, inclusion, relationships) may be even more important than health improvements per se [40]. Independence, psychological wellbeing, social relations, standard of living, and social activities are examples of factors that strongly influence older people’s quality of life [46]. As the concept of quality of life is more multi-dimensional for the elderly people and it is not only defined by health status, it should be measured by taking into account a broad range of dimensions, not only health status and/or functionality [45]. As the outcomes measured by QALY do not include non-health dimensions, overall social welfare is not actually assessed, but only overall population health [14]. A single preference-based instrument of QoL measurement, which could incorporate health status as well as other aspects of quality of life which are important for older people has not yet been constructed [40, 45].

### How can these issues be solved?

Some authors of the included publications indicated various modifications that could improve the QALY method. These changes could potentially be used to solve or at least to mitigate the effects of problems with using QALY for the older population. The summary of these proposals is presented in Table 3.

**Table 3:** The solutions of indicated problems suggested in the studies

Type of problem	Potential solution	Remarks
Lower QALY caused by lower LE	Introducing “end-of-life” rule (higher valuation of gained years in the case of terminal illnesses with no more than 2 years to live) [25].	When the EOL rule is implemented, age discrimination can only emerge under very rare conditions [25].
	Introducing discounting: gains of life years are diminished when they occur in the distant future [18].	Discounting greatly reduces the problem, and may make it almost negligible [18].
Equal value of QALY regardless of age	To adjust the results of QALY analysis to better match general public opinion by introducing age-correction (age weighting of QALY) [28, 30–33, 35, 37].	The problem is too complex to be solved by age-weights [31]. Research mainly focused on life-saving interventions may wrongly interpret social preferences [31]. Future research should be conducted before introducing such a solution, also combining age with other factors like gender or socio-economic status [30, 33]. More equitable distribution will cause lower efficiency [37]. Such weights may become arbitrary and give the possibility of abuse [37].
Lower gains in QoL possible for older people	Determining different thresholds of QALY accepted for financing for different age groups [40].	Not a sufficient solution if all dimensions important for older people’s QoL are not taken into account at the same time [40].
	Calibration of health state valuation to best attainable health prospect [41].	No motivation for seeking methods to improve a health state level which is deemed “normal” [41].
QoL measure instrument inadequacy	Developing a proper age-specific preference-based indicator for QoL measurement [40, 46].	
Health-related QoL measured regardless of age; no beyond-health QoL aspects taken into account	Using EQ-5D in combination with another instrument suitable for older people (e.g. ICECAP-O or ASCOT) [45].	There is no single existing measure that could assess QoL in a sense broad enough for older people [45]. Further research is needed to identify relevant attributes of health-related QoL for different age groups [38].
	Developing a new, age-specific measure of QoL, targeted at older people, based more on capability than functioning and preference-based utility [40, 46].	
	To allocate budget separately for different levels of care and beneficiaries’ age, using appropriate assessment criteria [26].	

Authors’ indication of a potential solution does not always mean that they consider it a recommendation

## Discussion

The QALY method can be used to help make a decision regarding which treatment/health activities should be provided to a given group of people or to which group of people care should be provided. The latter is much more problematic when it requires comparing the health gains in different age groups, especially if one of them is the older population. Our review showed an abundance of literature in which problems related to the use of QALY for older people are mentioned. These problems can be classified into six general categories, described in the article: (1) lower life expectancy in the old population causes lower QALY gains; (2) an equal value of one QALY is used regardless of age; (3) a poorer average health state causes lower QALY gains; (4) inadequate quality of life (QoL) measurement instruments; (5) attributes of QoL used regardless of age; and (6) no beyond-health QoL aspects taken into account. Although the problems seem to be clearly defined and quite well recognized, the potential solutions are much more ambiguous. Numerous authors emphasize the need for further research and consensus building activities in order to define recommendations.

The maximization of QALY does not offer the best solution to resource allocation problems, but even if QALY is not an ideal measure, this method might be seen as the best available and does have a lot of supporters [19, 20, 36]. QALY only indicates the option with the largest gain in population health, but there is no indication of which decision brings the highest overall social value [14]. Due to many limitations, QALY may be seen as not fully suitable for assessing health outcomes for older people, and its use is controversial. Many different ways to mitigate the problems that come with using QALY have been proposed (Table 2), but finding a widely accepted solution is a very difficult task.

There are different points of view and opinions regarding the possible ageist bias of QALY: one says that QALY discriminates against older people, the other says that it does not discriminate. Some experts even argue that QALY rather discriminates against young people and is not ageist enough [20, 30]. In QALY definition, the age of the person under consideration is not directly taken into account and this can, on first sight, be seen as a form of equality [29]. However, as presented above, after deeper analysis, many issues with equality may be identified.

Achieving allocative efficiency may not be socially favourable. The method using QALY assumes the primacy of health maximization; however, societal preferences demonstrate that other characteristics may affect priority setting [28, 35]. Some research indicates that due to general public opinion, especially in the case of life-saving/emergency treatment, younger people should receive priority over older people (e.g., [30, 32, 47, 48]. However, for treatment which improves quality of life rather than saving life, no such priorities are given [49]. What is interesting is that the opinion regarding valuing f younger people’s health more than older people’s does not change with age – younger and older people’s opinions are similar [30, 32, 48]. So in some cases, and to some extent, lower QALY gain in older age can be in line with social values, focusing health programmes on children and younger people [14]. Looking at social preferences, it can be stated that a bias may also occur sometimes in favour of older people, and health gains should be weighted, but rather giving lower weights to benefits in older age (e.g. [24, 26, 31, 34, 49]. Various possible reasons for valuing the health of the young more than that of the old can be indicated: (1) the impact of health in younger age on the whole development of an individual (health problems can have more than immediate consequences, but also future consequences); (2) more responsibility for others (e.g., for children); and (3) younger people can be seen as more valuable for society (productivity ageism) [9]. Even if we agree that in some cases the young should have some priority in receiving health care over the old, the problem gets more serious when it is necessary to set how much more priority should be given. Assigning age-weights may be seen as the best solution to, in a way, integrate efficiency and equity consideration. But such weights may become arbitrary and create the possibility for abuse, rather than achieving the desired results [37].

One important step in the QALY calculation is the valuation of QoL, often indicated as a problematic process. The perception of QoL in this case is usually limited to health aspects, but there is an ongoing discussion regarding the need for a broader approach to this quality, focused more on overall “well-being”, in order to better capture the full benefits of activities [7, 50]. An overly narrow understanding of the QoL may lead to underestimations of effects, especially in older populations. Developing a new measure, containing more than just health-related attributes, would make it possible to compare the full value of interventions, which would improve QoL by focusing impact on more than only health, which is often important with the older people [51].

Our review has provided a systematic overview of the literature related to the problems (and potential solutions to those problems) of applying the QALY measure to assess interventions for older people. To the authors best knowledge, it is the first study of this kind. Yet, it is not free from limitations. Due to feasibility of the work, we have used only two databases for the initial search, including full texts solely in English. This review has been limited to publications regarding the problems of using QALY for the older population. However, many issues which occur in the case of people with disabilities or in the last years of life may be similar or even identical, so further analysis should take into account more broadly defined groups of people in order to identify more potential problems. Additionally, some more issues regarding the analysed problems could possibly be found by directly checking studies on the QoL measurement methods typically used in QALY calculation (e.g., the Standard Gamble or Time Trade-off). The portions of publications concerning them may not directly point to QALY and, therefore, may not have been found in this search. Despite these limitations we believe we have provided a comprehensive overview and analytical framework for further investigations.

Life expectancy is still increasing, the health expectations of older people are changing, and so is the perception of older people in society. So, more current research is needed to better understand the health needs of older people and how to be more efficient in meeting them. Considering equity in allocation decisions, especially in the face of scarce resources, is a really challenging task, as health-care distribution will always include some degree of discrimination [19]. The concept of equity is very multi-dimensional, may be viewed subjectively, and can indicate inefficient solutions. To maintain the balance between efficiency and equity, issues connected with age-based rationing should be widely discussed.

## Conclusions

QALY is the most used measure of health effects and has been widely recommended so far, but this does not mean that nothing in this methodology can be improved. This review clearly shows that many problems of different types are connected with using QALY for the older population, but there is no consensus as to whether QALY discriminates against older people or not – one’s opinion regarding this issue depends strongly on accepted principles, particularly one’s approach to equity and how one understands fairness. QALY should be used for the older population with caution and further research is needed to improve methods in order to help make the best allocation decisions.

## Data Availability

Full data and material available upon request.

## Abbreviations

CUA: Cost-utility analysis
LE: Life expectancy
QALY: Quality Adjusted Life Year
QoL: Quality of life: YoL: years of life
